# Why Do Vulcanite Plates Cause Irritation of the Mouth in Some Cases?

**Published:** 1868-01

**Authors:** R. P. Nevill


### ARTICLE IV.

### *Why do Vulcanite Plates cause irritation of the mouth in some cases?*

By R. P. Nevill, D.D.S.

This question was discussed at the last meeting of the American Dental Convention without any definite solution, and without any remedy being suggested, save the exchange of gold for the vulcanite plate. Some who engaged in the discussion supposed it to be caused by the too close fitting of the plate. If this be true why does it not occur in all cases where the plate fits closely, and why does it occur in other cases where the plate fits loosely? Others accounted for it on the hypothesis that the rubber being a poor conductor, maintains continuously the same temperature. Why then does it occur in some cases where there is little tendency to inflammation, and fail to show itself in other cases where there is undoubted evidence of an inflammatory diathesis? Or why does it occur in some cases very soon after the insertion of the plate, and never in others after years of wear? Another supposed it to be caused by the mechanical closure of the mucous follicles. Has the same objection ever been urged against Cheoplasty, which is said to make as close a fit as rubber? Or are there any such unfavorable results attending the use of the aluminum base, which is said to be nearer absolute perfection with regard to fit than the vulcanite? There was one more suggestion offered as a solution to the question, viz.: that the rubber being more or less porous, absorbed the fluids of the mouth, these latter becoming vitiated and causing the soreness in question. This would seem to be a rational conclusion if it only occurred in those cases where the plate had been worn for a considerable length of time and the patient been negligent with regard to keeping it clean. But how shall we account for soreness produced by wearing a rubber plate but a few days, and the most scrupulous attention paid to cleanliness?

Dentists are careful (or should be at least) to give their patients particular instructions to keep their plates clean, and if some fail to carry out these instructions they may expect sore mouths, though we are not certain that it will be confined alone to those who wear rubber plates. Some months ago I inserted a full upper denture on rubber for a lady, in excellent health. The fit was remarkably good and she wore them with great satisfaction for a few days. In three or four days she returned complaining of soreness all round the edge of the plate. Supposing it to be caused by the plate extending too high on the ridge and too far back on the palate, I filed and scraped it at the points mentioned, and advised her to abstain from wearing it until the soreness disappeared. She returned after a short time again complaining of her mouth being sore from wearing the plate, and, to use her own language, "the parts seem to be greatly wearied and almost completely exhausted." There was now apparent more or less inflammation over the entire arch, and I was unable to account for it, never having met with a similar case. The plate being rather thin and weak and not vulcanized quite enough, I determined to do the work over, hoping by a new trial to obtain a better result. After leaving out the old plate for about a week or more, I obtained a new impression of the mouth and constructed a new set. I was careful to make it sufficiently low on the ridge, and to prevent it from encroaching upon the soft palate. From some cause or other I failed to get as good a fit in this as in the first set. The plate moved laterally, but at the same time required considerable force to detach it ; yet it was evident that adaptation was not so perfect as before. At this stage of the operation I was reminded of a remark made by the patient and I considered the whole difficulty, attending the first plate, solved.

When she first complained of the set, she said she thought if it did not fit so "tightly" it would not cause her mouth to become sore. Now I thought this loose fit the very *desideratum*, and adjusted the set in my patient's mouth with a considerable air of triumph. But alas how often do our most sanguine expectations fall to the ground. I saw the patient again after a few days, and she said that her teeth were doing well, except that her mouth was becoming sore again. I did not consider this doing so well, and advised her to leave them out until the soreness disappeared. This advice she followed some time, and now experiences no inconvenience from wearing the set.

Soon after this I met with the patient of another dentist, who was wearing a full upper set on rubber inserted about eighteen months previous. She informed me that her mouth became very irritable soon after the insertion of the plate, but that it recovered gradually until now she wears the set with greater comfort, although a slight degree of soreness yet remains. Due regard has been paid to cleanliness, and there is no offensive odour about it.

Had she laid the plate aside from time to time, and not persisted in wearing it when her mouth was in an irritable condition, she would doubtless have been well long before this ; for in this case the fit was very good. A short time since I inserted a full upper set for a lady, and so easily is she nauseated, that the simple presence of the set in her mouth made her very sick ; this, however, soon wore off but, in less than four days she complained of her mouth becoming irritable, not on account of the plate irritating the parts mechanically, but from the whole mucous membrane of the mouth becoming sore, and the soreness extending over the soft palate and into the throat ; the parts remaining in this condition to the present time. The fit of the plate is very good, and the patient is in remarkably good health, living at one of the public watering places in South Alabama. I advised her to persevere in wearing the plate at intervals in the hope that the trouble will soon entirely disappear, and also recommended the use of an astringent mouth wash.

Now I ask, for I write for information alone, why does irritation of the mouth attend the wearing of rubber plates in certain cases and what is the remedy? May it not be from some peculiar idiosyncracy preexistent in the patient, requiring however, the wearing of this peculiar work to develop the symptoms complained of; and may not this constitutional peculiarity be overcome by persistence in wearing the plate, allowing a sufficient interval from time to time for at least a partial subsidence of the irritation, together with the use of a good mouth wash to assist in allaying the inflammation? Or shall we as some have suggested, condemn the use of rubber altogether? And what then? Shall we return to the gold work, which never made s good aa fit as rubber and at the same time gave less satisfaction to our patients and is far more expensive? Or shall we adopt generally the continuous gum work, which with all of its many virtues, has also some faults? Or shall we try aluminum, a more recent material, knocking for admittance into our laboratories, and possessing, it is claimed, qualities that are not found in any other substance, before offered to the profession?

---

### ARTICLE V.

### *A Dislodged Tooth Replaced.*

